# The outer membrane protein, OMP71, of *Riemerella anatipestifer,* mediates adhesion and virulence by binding to CD46 in ducks

**DOI:** 10.1186/s13567-024-01393-9

**Published:** 2024-10-15

**Authors:** Yanhua Wang, Sen Li, Congran Ning, Rongkun Yang, Yaxin Wu, Xu Cheng, Jike Xu, Yi Wang, Fei Liu, Yang Zhang, Sishun Hu, Yuncai Xiao, Zili Li, Zutao Zhou

**Affiliations:** 1 College of Veterinary Medicine, Huazhong Agricultural University, Wuhan, China; 2 State Key Laboratory of Agricultural Microbiology, Huazhong Agricultural University, Wuhan, China; 3 Key Laboratory of Preventive Veterinary Medicine in Hubei Province, Huazhong Agricultural University, Wuhan, China; 4 Hubei Hongshan Laboratory, Huazhong Agricultural University, Wuhan, China

**Keywords:** *Riemerella anatipestifer*, CD46, OMP71, adhesion, virulence

## Abstract

The *Riemerella anatipestifer* bacterium is known to cause infectious serositis in ducklings. Moreover, its adherence to the host’s respiratory mucosa is a critical step in pathogenesis. Membrane cofactor protein (MCP; CD46) is a complement regulatory factor on the surface of eukaryotic cell membranes. Bacteria have been found to bind to this protein on host cells. Outer membrane proteins (OMPs) are necessary for adhesion, colonisation, and pathogenicity of Gram-negative bacteria; however, the mechanism by which *R. anatipestifer* adheres to duck cells remains unclear. In this study, pull-down assays and LC–MS/MS identified eleven OMPs interacting with duck CD46 (dCD46), with OMP71 exhibiting the strongest binding. The ability of an *omp71* gene deletion strain to bind dCD46 is weaker than that of the wild-type strain, suggesting that this interaction is important. Further evidence of this interaction was obtained by synthesising OMP71 using an *Escherichia coli* recombinant protein expression system. Adhesion and invasion assays and protein and antibody blocking assays confirmed that OMP71 promoted the *R. anatipestifer* YM strain (RA-YM) adhesion to duck embryo fibroblasts (DEFs) by binding to CD46. Tests of the pathogenicity of a Δ*omp71* mutant strain of RA-YM on ducks compared to the wild-type parent supported the hypothesis that OMP71 was a key virulence factor of RA-YM. In summary, the finding that *R. anatipestifer* exploits CD46 to bind to host cells via OMP71 increases our understanding of the molecular mechanism of *R. anatipestifer* invasion. The finding suggests potential targets for preventing and treating diseases related to *R. anatipestifer* infection.

**Supplementary Information:**

The online version contains supplementary material available at 10.1186/s13567-024-01393-9.

## Introduction

Duck infectious serositis is an acute contagious sepsis caused by *Riemerella anatipestifer* (*R. anatipestifer*, RA). This acute illness primarily infects ducks, geese, and other waterfowl through the respiratory tract or skin injury. While infected ducklings can have a mortality rate as high as 75%, the virus often presents as chronic or asymptomatic in adults [1, 2], making it difficult to detect and eradicate. Reports in recent years have indicated an increase in *R. anatipestifer* infections among domestic poultry in China, affecting broilers, laying hens, and breeder hens [3]. The capsule [4], lipopolysaccharide [5, 6], T9SS [7, 8], outer membrane proteins (OMPs) [9] and gelatinase [10] of *R. anatipestifer* are virulence factors that promote its survival in the host’s bloodstream. However, the pathogenesis of R. anatipestifer is complex, involving both the bacteria and the host. Little is known about the mechanism of infection, particularly the details of the adhesion and invasion of host cells.

Most OMPs in Gram-negative bacteria have a β-barrel structure, with an even number of β-chains arranged in parallel on the membrane in reverse order. The chains are connected by large, extended loops on the outer side of the cell wall and by short loops on the periplasmic side [11, 12]. The bacteria can better cope with diverse environments with this highly stable structure. The extracellular loops are in direct contact with the environment and have an important pathogenic role in host cell adhesion [13, 14], invasion [15], virulence [16], and immunogenicity [17]. Omp76, OmpH, OmpA, and other OMPs [9, 18, 19] have been found to be virulence factors of *R. anatipestifer*. OmpA is known to mediate adhesion to host cells [20], which makes it a good candidate antigen for an *R. anatipestifer* vaccine [21].

CD46, also known as membrane cofactor protein (MCP), is a type I transmembrane glycoprotein that acts as a complement regulator [22, 23]. The CD46 protein also serves as a cofactor for the plasma serine protein factor I (FI), which causes proteolysis of C3b and C4b deposited on the eukaryotic cell surface [24]. Human CD46 is widely expressed on the surface of all cells except red blood cells. The protein consists of four isomers, which are produced by selective splicing of a single 46 kb gene [25, 26]. It is also expressed in chickens and can be detected in most organs and tissues except for serum [27]. CD46 is considered a pathogen magnet because it can interact with a variety of pathogens [28], including *Neisseria gonorrhoeae* and *Neisseria meningitidis*, *Streptococcus pyogenes*, *Escherichia coli*, herpesvirus 6, and the measles virus [29–34].

In this study, we expressed dCD46 as a bait protein to capture the OMPs of RA-YM. We found that OMP71 bound strongly to dCD46. Furthermore, the interaction between the two proteins promoted the adhesion of *R. anatipestifer* to duckling cells. Pathogenicity testing found OMP71 to be a key virulence factor of RA-YM. These findings provide a scientific basis for better understanding the mechanism of pathogenesis and adhesion of *R. anatipestifer*.

## Materials and methods

### Source of animals and housing conditions

One hundred and eighty one-day-old Cherry Valley ducks were purchased from the Wuhan Yongsheng Duck Farm (Hubei, China) and housed at the laboratory animal centre of Huazhong Agricultural University. Throughout the study period, 10 ducks were housed in a cage in a temperature-controlled environment. The ducks were exposed to light for 12 h each day and had access to unlimited antibiotic-free food and water.

### Ethics statement

All animal experiments were approved by the Huazhong Agricultural University animal centre and carried out per the recommendations in the Guide for the Care and Use of Laboratory Animals from the Research Ethics Committee, Huazhong Agricultural University, Hubei, China (approval no. HZAURAB-2024-0008). All procedures involving animals were performed following the ethical standards of the institution or practice at which the studies were conducted.

### Plasmids, bacterial strains, and eukaryotic cells

The bacterial strains and plasmids used in this study and their corresponding sources are listed in Table 1. RA-YM and the deletion strain were cultured in tryptic soy broth (TSB) (Becton, Dickinson Co., Franklin Lakes, USA) or Giolitti-Cantoni broth (GCB) at 37 ℃ in a 5% CO_2_ incubator. Newborn bovine serum (NBS) was added to tryptic soy agar (TSA) plates (Becton, Dickinson Co.) and Giolitti-Cantoni agar (GCA) plates to a final concentration of 3%. *E. coli* strains DH5α, DH10Bac, BL21 (DE3), Rosetta (DE3), and X7213 were cultured in Luria–Bertani (LB) broth or grown on LB plates at 37 °C [35].

**Table 1:** **List of strains, plasmids and cells used in this study**

Strain or plasmid	Characteristics	Sources
*Riemerella anatipestifer*		
RA-YM	*Riemerella anatipestifer* wild-type strain, serotype 1	Laboratory preservation
RA-YM Δ*omp71*	*omp71* gene deletion mutant strain, Spec^R^	This study
RA-YM CΔ*omp71*	Complemented Δ*omp71* strain, Spec^R^, Erm^R^	This study
*Escherichia coli*		
*E. coli* DH5α	F-φ80 *lacZΔM15* Δ(*lacZYA-argF*) *U169 endA1 recA1 hsdR17*(rk^−^, mk^+^) *supE44* λ^−^ *thi-1 gyrA96 relA1 phoA*	Invitrogen
*E. coli* DH10Bac	F^−^ *mcrA* ∆(*mrr-hsdRMS-mcrBC*) ϕ80*lacZ∆M15 ∆lacX74 recA1 endA1 araD139* ∆ (*ara, leu*)7697 *galU galK* λ^−^ *rpsL nupG* /pMON14272/pMON7124	Gibco
*E. coli* BL21 (DE3)	*E. coli* B F^−^ *dcm ompT hsdS* (rB- mB-) *gal* [*malB* +] K-12 (λS)	Invitrogen
*E. coli* Rosetta (DE3)	*F- ompT hsdS*_*B*_*(r*_*B*_^*−*^*, m*_*B*_^*−*^*) gal dcm(DE3) pRARE(argU, argW, ilex, glyT, leuW, proL) (Cam*^*R*^*)*	Sigma
*E. coli* X7213	*thi-1 thr-1 leuB6 glnV44 fhuA21 lacY1 recA1* RP4-2-Tc: Mu λ *pir1 asdA41 zhf-2*:Tn*10*	[8]
Plasmids		
pMD18-T	TA cloning vector	Takara
PFastBac^TM^1	Baculovirus expression vector	Gibco
pCold-TF	Expression vector, Amp^R^	Takara
pRES-JX-Erm	Shuttle vector, Cmr^R^, Spc^R^, Erm^R^	[8]
pET-16b	Expression vector, Amp^R^	Sigma
Cells		
Sf9 insect cells	Sf9 cells were derived from the ovaries of *Spodoptera frugiperda*	Thermo Fisher Scientific
DEF cells	Duck embryonic fibroblasts (DEFs) were isolated from the embryo of a normal duck, immortalised and subcultured	ATCC

*Spodoptera frugiperda* (Sf9) insect cells and duck embryo fibroblasts (DEFs) were maintained in our laboratory. All cells were passaged as stated. The Sf9 insect cells were cultured in Sf-900TM II SFM (Gibco, Grand Island, USA) with shaking incubation (140 rpm) at 28 ℃. When the number of cells reached 2 × 10^6^/mL, they were subcultured at 1:2–1:4 according to the cell state. DEFs were cultured in Dulbecco’s modified Eagle’s medium (DMEM, Gibco, MD, USA) with 10% foetal bovine serum (FBS, Gibco) at 37 ℃ and 5% CO_2_. The cell medium was changed daily, and the cells were subcultured after achieving 80–90% confluence. Sf9 and DEF cells (2 × 10^6^/mL) were suspended in cryopreservation solution (90% FBS + 10% DMSO), frozen, and stored in liquid nitrogen.

### Recombinant baculovirus construction and purification of eukaryotic CD46

Total RNA was extracted from the fresh livers of healthy thirty-day-old Cherry Valley ducks and used for cDNA production by reverse transcription with the Evo M-MLV RT kit (Accurate Biology AG, Changsha, China). The duck *CD46* gene [27] (1227 bp) was engineered with the Strep-tag II peptide sequence for one-step affinity purification of the fused protein. The tagged gene was cloned into the pFastBac1 plasmid and transformed into *E. coli* DH10Bac for transposition.

Three generations of blue-white screening obtained the recombinant baculovirus Strep-dCD46 plasmid. The Strep-dCD46 plasmid was transfected into Sf9 cells and amplified to the P4 generation, where the virus titre reached 10^9^ pfu/mL. The Strep-dCD46 recombinant protein was expressed to a high level, after which it was extracted and purified using previously described methods [9].

### Fractionation of bacterial OMPs

An RA-YM inoculum was transferred into a TSB medium at a ratio of 1:100 and allowed to grow at 37 ℃ with shaking overnight. The bacteria were collected by centrifugation at 8000 × *g* for 10 min at 4 °C, resuspended in pre-chilled 100 mM Tris–HCl (pH 7.3), and disrupted in a high-pressure (1200 bar) apparatus (Juneng Nano-Bio Technology Co., Ltd., Guangzhou, China) at 4 ℃. Bacterial debris was removed by centrifugation at 12 000 × *g* for 15 min at 4 ℃.

The supernatant was centrifuged in an Optima XPN-100 (Beckman Coulter, Indianapolis, USA) at 100 000 × *g* for 1 h at 4 ℃ to collect the membrane fraction. The membranes were disrupted by stirring for 30 min at 37 °C in 1% sodium lauroylsarcosine. The suspension was again centrifuged at 100 000 × *g* for 1 h at 4 °C, and the pellet was dissolved in phosphate-buffered saline (PBS). The protein concentration in the cell membrane fraction was determined using a BCA protein assay kit (Bio-Rad, Hercules, USA) and stored at −80 °C.

### Western blotting

The presence of OMPs on RA-YM interacting with dCD46 protein was verified by far-Western blotting. The OMPs were extracted from RA-YM, and equal amounts were separated using SDS-PAGE. They were then electro-transferred to a PVDF membrane. The blot was blocked with 5% skimmed milk for 2 h, washed thrice with TBST (containing 0.1% Tween-20), and incubated with 0.2 mg of Strep-rdCD46 protein overnight at 4 ℃. After washing, the blot was sequentially incubated with mouse anti-Strep-II-tagged mAb (1:4000 dilution) and HRP-goat anti-mouse IgG (H + L) (1:10 000 dilution) (ABclonal). It was visualised by enhanced chemiluminescence (ECL) (Bio-Rad, Hercules, USA).

### Protein pull-down assays

The purified Strep-dCD46 recombinant protein (100 μg) was incubated with 50 μL of Magrose Strep-Tactin beads (Solarbio, Beijing, China) in 750 μL binding buffer (10 mM Tris–HCl, 150 mM NaCl, 1 mM EDTA, pH 8.0) for 2 h at 4 ℃; it was then washed thrice with 750 μL of binding buffer. The Strep-Tactin beads, both those coupled with Strep-dCD46 and those not coupled, were incubated with the extracted RA-YM OMPs (500 μg) at 4 ℃ for 4 h. The beads were washed five times with wash buffer (10 mM Tris–HCl, 150 mM NaCl, 1 mM EDTA), and bound proteins were eluted in elution buffer (2.5 mM desthiobiotin in binding buffer, pH 8.0).

The isolated proteins were analysed using mass spectrometry (Novogene, Beijing, China). Pull-down assays verified the direct combination of Strep-dCD46 and His6-OMP71. In His pull-down, His_6_-OMP71 and His_6_-TF (negative control) were coupled with IDA-Ni beads to fish Strep-dCD46 protein, respectively. In the Strep pull-down, Magrose Strep-Tactin Beads coupled with Strep-dCD46 were used to fish His_6_-OMP71 and His_6_-TF proteins, respectively. The proteins stripped from magnetic beads were then validated using mouse anti-His-Tag mAb (1:4000 dilution), mouse anti-Strep-II-tagged mAb (1:4000 dilution) and HRP-goat anti-mouse IgG (H + L) (1:10 000 dilution) (ABclonal).

### Mass spectrometry identification of pull-down assay proteins

Mass spectrometry at Novogen (Beijing, China) analysed the protein samples from the pull-down assay. The acquired data from triplicate MS runs for each sample were combined and searched against ASM1929781v1 (National Center for Biotechnology Information). Proteome Discovery 2.2 software was used to search the database, with the following parameters set: 10 ppm for the mass tolerance of precursor ions and 0.02 Da for fragment ions. Peptide spectrum matches (PSMs) with confidence > 0.99, and proteins containing at least one unique peptide were retained to improve the quality of the analysis results. Peptides and proteins with FDR > 1% were removed. Individual protein function was annotated by Interproscan software, with the identified proteins classified by protein family.

### Cloning, expression, and purification of candidate OMP genes

Using the RA-YM genome as a template, the candidate outer membrane protein genes of *R. anatipestifer* were amplified using the primers specified in Table 2. The protein genes were purified and cloned into the pCold-TF plasmid. The plasmids, pCold-TF, pCold-TF-*raga*, pCold-TF-*omp71*, pCold-TF-*susc*, pCold-TF-*port*, pCold-TF-*clp*, pCold-TF-*pnplas*, pCold-TF-*omp β-brl*, pCold-TF-*hp*, pCold-TF-*tbux*, pCold-TF-*duf4353*, and pCold-TF-*opro* were transformed into *E. coli* Rosetta (DE3) for recombinant protein expression.

**Table 2:** **List of oligonucleotide primers used in this study**

Primer	Sequence (5′-3′)	Purpose
P1	CCGCTCGAGGCCACCATGGGATCATGGAGCCACCCTCAGTTCGAAAAGATGCAGAGCTTTCCCGTTGGAAC	To amplify the dCD46 fragment
P2	CCCAAGCTTTCACTTTTCGAACTGAGGGTGGCTCCAGGAACCACCGCCTCCTTTACAGCTCGTATATTTGGCAG	
P3	GTTTTCCCAGTCACGAC	To identify the Bacmid-dCD46
P4	CAGGAAACAGCTATGAC	
P5	CGCCATATGATGAATGTGAAATTAAGGGTGCTTAC	To construct the pCold-TF-raga
P6	CCGCTCGAGAAAACCAACATTCAATCCTAAAGCG	
P7	CCGCTCGAGATGATATTTTTAATAAAAACTTTCGGAGG	To construct the pCold-TF-omp71
P8	CCCAAGCTTAAAATTAGTTTTTAAAGTAGTTAAAAAATAC	
P9	CGCCATATGATGAAGAAATTAACAACAAGTGTTTTAGC	To construct the pCold-TF-susc
P10	CCGCTCGAGAAATTTAAATGTTACATCTATACCTATATC	
P11	CGCCATATGGAGCTCGGTACCCAAATCAATATTCAG	To construct the pCold-TF-port
P12	CCCAAGCTTTTCAAAAATATAATTAAGTGTTACAC	
P13	CGCCATATGCAGGTAACTACAGGAAGTATGACTGG	To construct the pCold-TF-clp
P14	CCGCTCGAGGAAAGTATATTTAAATCCTAATACAGCAC	
P15	CCGCTCGAGATGCTTATGAAAAAAGTATTATGGCTG	To construct the pCold-TF-pnplas
P16	CCCAAGCTTAAACCAATGTCCTAATACGACGTTAA	
P17	CCGCTCGAGCAATCCAATAAAGAAGACTCTAAAG	To construct the pCold-TF-omp β-brl
P18	CCCAAGCTTTTTCAATCTATCTATTGTATTATCCGTAG	
P19	CCGCTCGAGCAGGATGTGTCTGTAATCAGGAATACG	To construct the pCold-TF-hp
P20	CCCAAGCTTAAATCTCCAACCGACTGTAAACAC	
P21	CCGCTCGAGGGAGGCTTTAGAGTTTCTTTGC	To construct the pCold-TF-tbux
P22	CCCAAGCTTAAAAGGGTTGTAAGATAGGCC	
P23	CGCCATATGCAAACCAGTATCTCTGGTAAAATTACC	To construct the pCold-TF-duf4353
P24	CCGCTCGAGTTTAAAATTAAACTTTACGGTAAACATC	
P25	CCGCTCGAGCAAGAAGTTAAAGTACCAGATACTATTATC	To construct the pCold-TF-opro
P26	CCCAAGCTTAATTCCTATTTCTACTTGAAATCTAGCG	
P27	CAAGCTTCTTCTAGAGGTACCATACAGCAAGACCCATTATTTATATTCTC	To construct the Δ*omp71*
P28	TTCGTTCCACTGTCGTTTGGCAGGTCTCCTG	
P29	CTTTTAAAACTACTGTATCCATATTTTATGAAAAAATC	
P30	GGAGAGCTCGATATCGCATGCGCCAGATACAATGTACTC	
P31	CCAAACGACAGTGGAACGAAAACTCACGTTAA	
P32	GGATACAGTAGTTTTAAAAGTAAGCACCTGTTATT	
P33 P34	CGCCATATGATGGTGAATATTTTGCAGTGATTTAG CGCCATATGTTAAAAATTAGTTTTTAAAGTAGTTAAAAAATACC	To construct the CΔ*omp71*
P35	ATGATATTTTTAATAAAAACTTTCGGAGGAGG	To identify the Δ*omp71* and CΔ*omp71*
P36	GTTTTTAAAGTAGTTAAAAAATACCTGC	
P37	GATTAACGGAAAAATACCAAATCAAGCGAT	
P38	GCTTTCACCTTTGAATATAGTCCTTCCCAC	

When the bacterial cultures reached an OD_600_ of 0.6–0.8, isopropyl-1-thio-b-D-galactopyranoside (IPTG) was added to a final concentration of 0.6 mM, and induction was continued at 16 ℃ for 18 h. The IPTG-induced cultures were centrifuged at 6000 × *g* at 4 ℃ for 10 min. The pellets were resuspended in binding buffer (20 mM Na_3_PO_4_, 500 mM NaCl, 30 mM imidazole, pH 7.4), and the cells were disrupted by three cycles at 1000 bar in a high-pressure homogeniser at 4 ℃. The supernatants were collected by centrifugation at 12 000 × *g* at 4 ℃ for 30 min, and the corresponding protein was purified on a His-Trap HP column (Cytiva AB, Uppsala, Sweden).

### Protein binding assays

Protein binding assays were performed as described by Singh et al. [36]. Purified His_6_-tagged recombinant proteins (700 ng per well) were coated on a 96-well plate at 4 ℃ overnight. The coating solution was removed, the plate was washed, blocking solution (20 mM Tris, 150 mM NaCl, 0.1% Tween-20, 0.5% BSA) was added, and the plate was then incubated at 37 ℃ for 2 h to reduce nonspecific binding. Subsequently, 1 μg of Strep-dCD46 protein was added to each well and allowed to bind at 37 ℃ for 1 h. Mouse anti-Strep-II Tag mAb (1:5000 dilution) and HRP-conjugated goat anti-mouse (H + L) antibody (1:7000 dilution) were added sequentially after 1 h incubation at 37 °C. After washing, 100 μL of tetramethylbenzidine (TMB) substrate for ELISA (GBCBIO Technologies, Guangzhou, China) was added to each well, and the plate was held in the dark at room temperature. Following the maximal colour change, 50 μL of stop solution (2 M H_2_SO_4_) was added to each well. The OD_450_ values were then measured using a Multiscan MK3 microplate reader (Thermo, Waltham, USA).

### Construction of the deletion strain Δ*omp71* and complement strain CΔ*omp71* of RA-YM

The 1044 bp spectinomycin-resistance (*Spec*) gene was amplified by polymerase chain reaction (PCR) using PIC333 plasmid as the template, while the 1200 bp left and right homology arms of the *omp71* gene were amplified using the RA-YM strain as the template. The three fragments were concatenated by overlapping PCR to construct the spectinomycin-resistance expression cassette containing the *omp71* gene deletion. The RA-YM Δ*omp71* was generated according to the method described by Liu et al. [37], and the CΔ*omp71* of RA-YM was constructed as described previously [7].

### OMP71 mediates the binding of dCD46 to *R. anatipestifer*

The wild-type (WT) strain and the *omp71* deletion mutant were grown to the logarithmic phase in TSB to test the binding of dCD46 to RA-YM. As an additional check of the specificity of CD46 binding, the recombinant expression vector pET-16b-*omp71* was constructed and transformed into *E. coli* BL21.

*E. coli* BL21 (DE3)/pET-16b and *E. coli* BL21 (DE3)/pET-16b-*omp71* were grown in LB to an OD_600_ of 0.6–0.8 followed by an additional incubation at 28 ℃ with 1 mM IPTG for 6–8 h to induce the expression of OMP71 on the surface. The bacteria (10^8^ CFU) were incubated with 100 μL of Strep-dCD46 protein at various concentrations (0–100 ng/μL) for 1 h at 37 ℃, then washed thrice with PBS containing 1% BSA. The bacteria were lysed, and protein was extracted and assayed by Western blot for CD46 using the relevant antibodies.

### Evaluation of RA-YM and RA-YM Δ*omp71* binding to duck embryonic fibroblasts

The RA-YM bacterial strain adherence capacity was compared with that of the *omp71* deletion mutant using a binding assay with DEF cells following a modified method of Li et al. [20]. Confluent DEF monolayers in 24-well plates were washed twice with sterile PBS (pH 7.4) to remove unattached cells. Log-phase RA-YM, RA-YM Δ*omp71*, and RA-YM CΔ*omp71* strain bacteria were added to each well in triplicate at a multiplicity of infection (MOI) of 100, and the plate was incubated at 37 ℃ under a 5% CO_2_ atmosphere. After 2 h incubation, the monolayers were washed thrice with sterile PBS to remove unbound bacteria. Adding 0.25% trypsin detached the adherent bacteria, and the digestion was terminated with DMEM containing FBS. The adherent RA-YM, RA-YM Δ*omp71*, and RA-YM CΔ*omp71* cells were serially diluted and spread on TSA plates containing 5% NBS for counting. For the test of invasiveness, the cells with adherent bacteria were washed thrice with PBS, followed by the addition of DMEM containing 100 μg/mL gentamicin. After incubation at 37 ℃ for 1 h, the cells were washed and digested as described above. In total, the experiments were performed three times.

For measuring the adhesion efficiency of the RA-YM bacterial strain, 1 × 10^9^ CFU/mL RA-YM and RA-YM Δ*omp71* were stained with 20 μM carboxyfluorescein diacetate succinimidyl ester (CFDA-SE) for 30 min at 37 ℃, then washed with PBS and centrifuged at 3000 × *g* for 10 min. The bacterial pellets were resuspended in DMEM for later use. The CFDA-SE-labelled bacteria were used for the cell adhesion assay, which was performed as described above. The fluorescence intensity was measured with excitation at 488 nm and emission at 518 nm using a Perkin Elmer EnSpire microplate reader. Fluorescence images were acquired with an Olympus IX-73 microscope. The data represents the average of three measurements.

### Production of antibodies to duck CD46 and OMP71

New Zealand white rabbits were immunised with 500 μg of purified His_6_-OMP71 or His_6_-dCD46, respectively, mixed with Freund’s complete adjuvant (FCA) (Sigma-Aldrich, Missouri, USA) and fully emulsified. On days 14 and 28 after the first immunisation, 300 μg of proteins and Freund’s incomplete adjuvant (FICA) (Sigma-Aldrich, Missouri, USA) were administered to the rabbits, and their antibody titres were measured using ELISA. After reaching the maximum Ab titre, blood was drawn from the carotid artery. It was allowed to clot and then centrifuged to obtain serum. The serum was tittered for the specific polyclonal antibody and stored in aliquots at −80 ℃.

### Competitive inhibition assays for OMP71 binding to duck embryo fibroblasts

To evaluate how much OMP71 contributes to *R. anatipestifer’s* adhesion to DEF cells, we performed competitive inhibition assays using purified OMP71 protein, anti-OMP71 polyclonal antibody, and anti-dCD46 polyclonal antibody. DEFs were seeded in 24-well plates, grown to confluence, and incubated with OMP71 protein (50 μg/well) or 20-fold diluted anti-dCD46 polyclonal antibody for 1 h at 37 ℃ in a 5% CO_2_ atmosphere.

In addition, cells and bacteria were treated with 50 μg BSA or the same dilution of negative rabbit serum as the controls. DEFs were washed thrice with PBS, and 5 × 10^7^ CFU/well RA-YM was added and incubated for 2 h under the same conditions. Non-adherent bacteria were removed by washing thrice with sterile PBS, and the adherent cells were detached from the DEFs with 0.25% trypsin and collected by aspiration and centrifugation. The RA-YM pellets were resuspended, serially diluted, and spread onto TSA plates for counting after overnight incubation.

To test the effect of blocking OMP71 on RA-YM adhesion to DEF cells, we incubated 10^9^ CFU RA-YM with 20-fold dilutions of anti-OMP71 polyclonal antibody and negative control serum at 37 ℃ for 30 min. This process was followed by a 2-h incubation with DEFs at an MOI of 100 at 37 ℃. Adherent cells were collected as described above, serially diluted, and spread on TSA plates. Colonies were counted after overnight incubation at 37 ℃.

### Assay for pathogenicity of the RA-YM Δ*omp71* deletion strain in ducklings

RA-YM, RA-YM Δ*omp71*, and RA-YM CΔ*omp71* were grown until they reached the logarithmic phase. They were centrifuged at 3000 × *g* for 10 min and washed thrice with PBS. As previously described for determining the LD_50_ of RA-YM in ducklings [7], twelve-day-old ducklings were injected into the flipper with either the wild-type strain or the *omp71* gene deletion strain at doses of 10^9^, 10^8^, 10^7^, 10^6^, and 10^5^ CFU per duckling. The mortality and morbidity of the ducklings were recorded for seven days after the challenge, with survival curves plotted and the LD_50_ values calculated.

Twelve-day-old ducklings were divided into three groups and injected with 0.2 mL (10^6^ CFU) of RA-YM, RA-YM Δ*omp71*, or an equal volume of PBS as a negative control to determine the effect of infection on blood and major organs. Tissue samples from the blood, heart, liver, spleen, and brain were collected from three ducklings in each group after 24 h and 48 h. The bacterial load in blood and tissues was determined using the same method described above [38]. At the same time, the above tissues were fixed, embedded, sectioned, and stained for microscopic evaluation of pathological changes [9]. Furthermore, to obtain serum, the blood samples collected at 24 h and 48 h after the challenge were incubated at 37 ℃ for 30 min and centrifuged at 1600 × *g* for 10 min. The expression levels of duck interleukin-6 (IL-6) and interleukin-8 (IL-8) were detected using an ELISA kit (Meimian Industrial Co., Ltd, Jiangsu, China).

## Results

### Expression of duck CD46 and screening of interacting OMPs of *R. anatipestifer*

**Figure 1:**
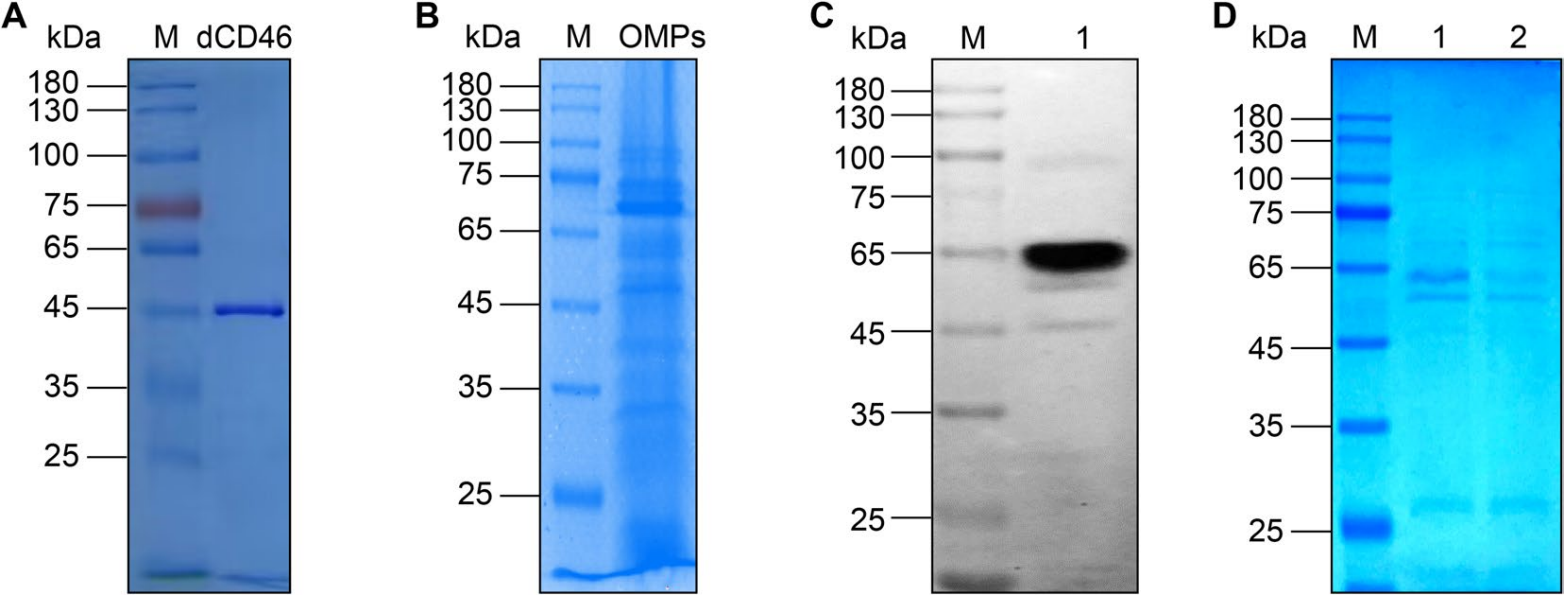
**Expression of duck CD46 and screening of interacting OMPs of *R. anatipestifer*. A** SDS-PAGE analysis of purified eukaryotic recombinant protein Strep-dCD46. **B** SDS-PAGE analysis of OMPs isolated from the RA-YM strain. **C** The RA-YM OMPs were shown to interact with dCD46 protein by far-Western blot. Lane M, molecular weight marker; lane 1, RA-YM OMPs interacting with purified dCD46 protein. Purified dCD46 protein was used as the primary binding protein to detect the interacting OMPs, and the Strep-tag II antibody was used for visualisation of the interaction. **D** The interaction of dCD46 protein with RA-YM OMPs by pull-down assay. Lane M, molecular weight marker; lane 1, Strep-Tactin beads coupled with Strep-dCD46 pull-down of RA-YM OMPs (dCD46-OMPs); lane 2, Strep-Tactin beads not coupled with Strep-dCD46 pull-down of RA-YM OMPs (OMPs). Three independent experiments were conducted, and a representative experiment is shown here.

The CD46 gene from the Cherry Valley duck was cloned into the pFastBac1 expression vector, and a Strep-tag II was fused to the dCD46 protein N terminus to detect and purify the recombinant protein. Western blot and indirect immunofluorescence assays were used to verify the expression of recombinant Strep-dCD46 protein in Sf9 cells (Additional file 1). Recombinant Strep-dCD46 protein was verified by SDS-PAGE (Figure 1A).

The extracted OMPs of RA-YM were identified by SDS-PAGE (Figure 1B). Subsequently, the interaction between the purified dCD46 protein and OMPs of RA-YM was verified using the far-Western blot technique (Figure 1C). To screen for the OMPs of RA-YM that can interact with dCD46 protein, we used purified dCD46 protein as bait to capture OMPs by pull-down assays (Figure 1D) and showed that the experimental group bound more OMPs.

### Determination of the RA-YM OMPs proteome

**Figure 2:**
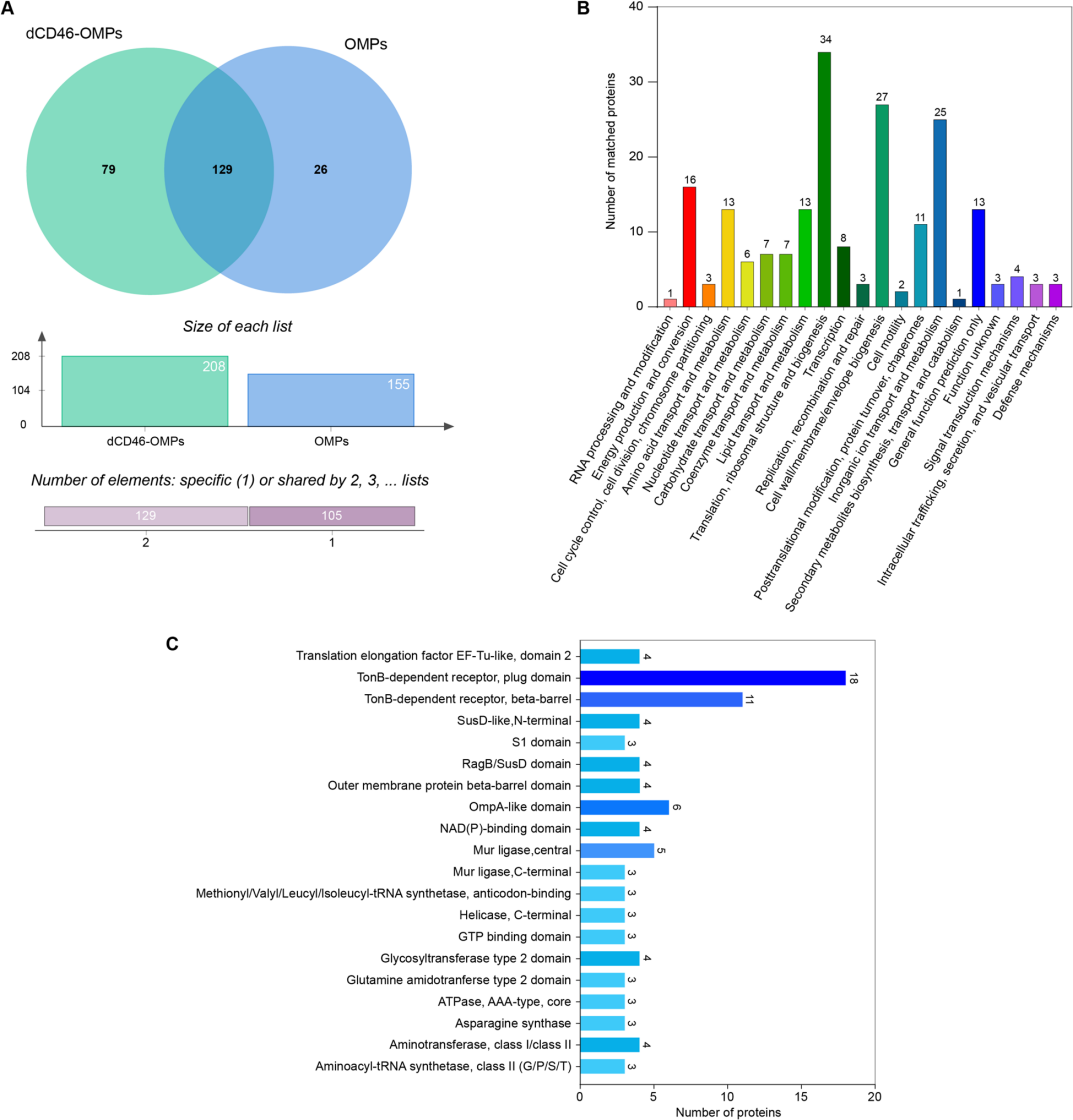
**Determination of the *R. anatipestifer* OMPs proteome. A** Venn diagram of Strep-Tactin beads coupled with Strep-dCD46 and Strep-Tactin beads not coupled with Strep-dCD46 interacting with *R. anatipestifer* OMPs. **B** COG analysis of *R. anatipestifer* OMPs interacting with dCD46 protein. **C** IPR analysis of *R. anatipestifer* OMPs interacting with dCD46 protein.

Mass spectrometric identification of OMPs bound to Strep-dCD46 beads (dCD46-OMPs) and bead-bound OMPs (OMPs) was performed. The results showed that 208 different RA-YM strain OMPs were bound by the Strep-dCD46 beads and 155 different OMPs bound to beads alone. The dCD46-OMPs group comprised 79 unique interacting proteins (Figure 2A), and a bioinformatics analysis was conducted on all of them. The Database of Clusters of Orthologous Genes (COG) annotated these proteins into 21 functional categories, including cell wall/membrane/envelope biogenesis, inorganic ion transport, and metabolism (Figure 2B). The domains of the proteins were annotated using Interproscan, which revealed mainly TonB-dependent receptors (Figure 2C). After removing non-membrane proteins using the UniProt website, the remaining proteins were screened according to the sum of the posterior error probability (PEP) scores, coverage, unique peptides, and the main functions. In total, 11 candidate proteins that interact with dCD46 were identified (Table 3).

**Table 3:** **Analysis of outer membrane proteins interacting with dCD46 by protein mass spectrometry**

Protein name	Accession	Sun PEP score	Coverage	Unique peptides	Main function
RagA	WP_214192532.1	10.836	8	7	SusC/RagA family TonB-linked iron transport receptors
OMP71	WP_004917785.1	1.534	2	2	TonB-dependent outer membrane cobalamin receptor protein
SusC	WP_014615125.1	8.88	5	4	SusC/RagA family TonB-linked monomeric catechols outer membrane receptor
PorT	WP_004918655.1	3.295	9	2	PorT family opacity protein and related surface antigens
CLR	WP_237190924.1	22.392	12	12	Carboxypeptidase regulatory-like domain-containing protein
PNPLAs	WP_014937791.1	2.13	3	2	Patatin-like phospholipase family protein
OMPβ-brl	WP_004920618.1	3.591	4	2	Outer membrane beta-barrel receptor proteins, mostly Fe transport
HP	WP_004918438.1	20.768	22	9	Outer membrane beta-barrel long-chain fatty acid transport protein
tubX	WP_004916211.1	13.364	14	6	Outer membrane protein long-chain fatty acid transport protein
DUF4353	WP_004917270.1	9.071	6	6	Outer membrane receptor for ferric coprogen and ferric-rhodotorulic acid
OprO	WP_004919636.1	6.118	9	4	Phosphate-selective porin

### Screening for *R. anatipestifer* OMPs that interact with dCD46

**Figure 3:**
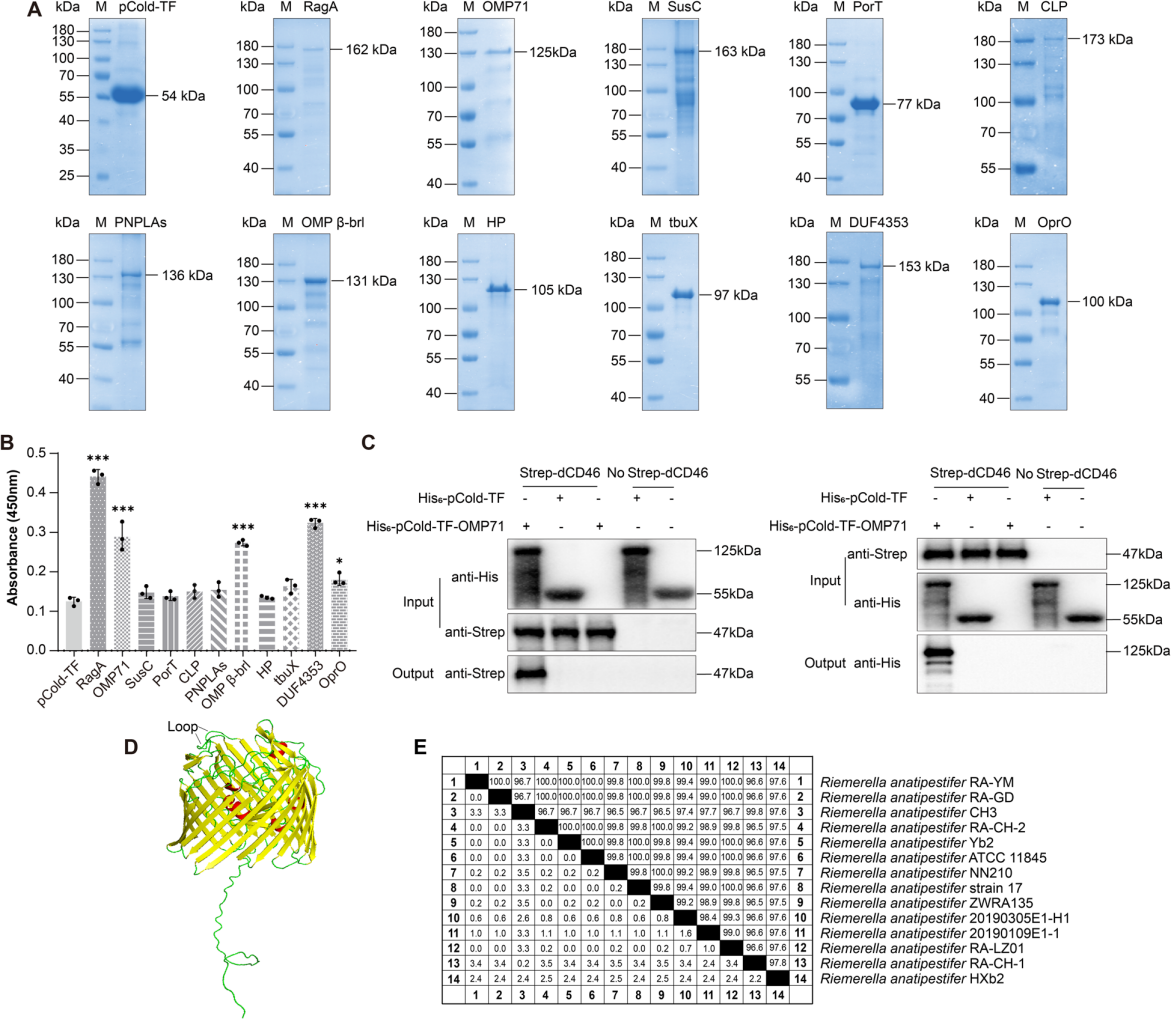
**Screening for *R. anatipestifer* OMPs that interact with dCD46. A** Analysis of His_6_-tagged recombinant RA-YM strain OMPs by SDS-PAGE. **B** Binding assays were performed to verify the binding of His_6_-tagged recombinant proteins to rdCD46 protein. **C** Analysis of His_6_-OMP71 binding to rdCD46 protein by pull-down. All data are the mean of three experimental replicates, and data were analysed by one-way ANOVA, with error bars representing standard deviations. *P ≤ 0.05; ***P ≤ 0.001. **D** The tertiary structure of OMP71 predicted by AlphaFold. Red, helix; yellow, β-strand; green, loop. **E** Homology analysis of OMP71 protein among different *R. anatipestifer* strains.

The RA-YM OMPs interacting with dCD46 are *raga* (KYF39_06585), *omp71* (KYF39_09665), *susc* (KYF39_03860), *port* (KYF39_03040), *clp* (KYF39_06470), *pnplas* (KYF39_05570), *omp β-brl* (KYF39_05875), *hp* (KYF39_03355), *tbux* (KYF39_06330), *duf4353* (KYF39_04665), and *opro* (KYF39_08185). His_6_-TF protein and His_6_-TF recombinant proteins were verified by SDS-PAGE (Figure 3A). We used a protein binding assay to assess the interactions between rdCD46 and RA-YM OMPs. His_6_-RagA, His_6_-OMP71, His_6_-OMP β-brl, His_6_-DUF4353, and His_6_-OprO all showed some differences in OD_600_ values compared with the control His_6_-TF (Figure 3B).

In this study, we first characterised OMP71 and how it interacts with dCD46, which was validated using His pull-down and Strep pull-down assays, as shown in Figure 3C. To better understand the screened proteins, we predicted their structure and assessed their conservation. This process found that the outer membrane protein (OMP71) comprises 622 amino acids and has a molecular weight of 71 kDa. Therefore, it was suitably named OMP71, and the gene *KYF39_09665* encoding this protein was named *omp71*. AlphaFold2 software, which predicts the tertiary structure of a protein, revealed that OMP71 is a β-barrel transmembrane protein composed of 22 antiparallel β-folds with 11 extracellular loops (Figure 3D). The homology of OMP71 protein in 14 typical *R. anatipestifer* strains was further analysed by MegAlign, with results showing the homology as 96.5–100% (Figure 3E).

### OMP71 of RA-YM binds to dCD46 protein

**Figure 4:**
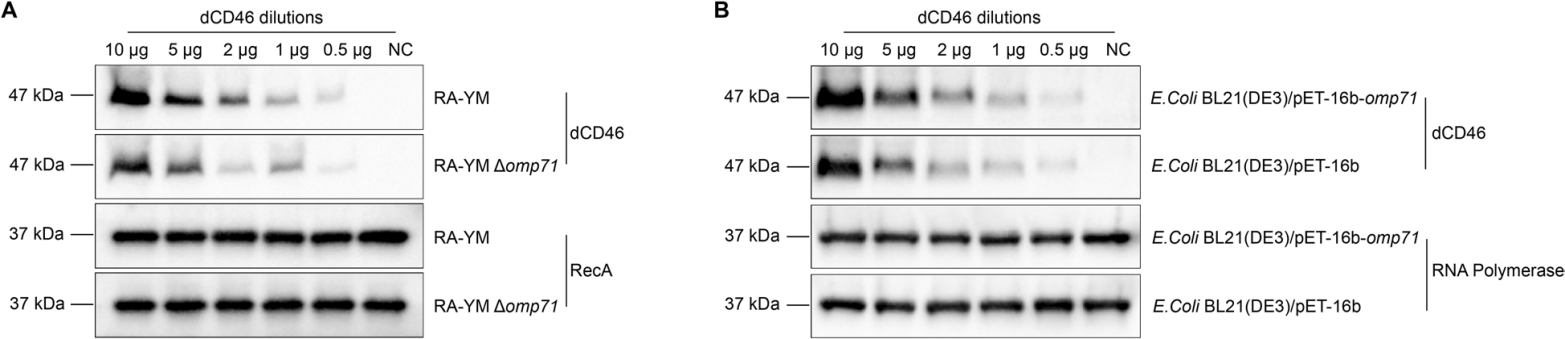
**OMP71-expressing RA-YM and *E. coli* strain binds to dC4BP. A** Western blot analysis of RA-YM wild strain and *omp71* gene deletion strain binding to purified dCD46 protein. **B** Western blot analysis of the binding of OMP71 expressed on the surface of *E. coli* and *E. coli* without OMP71 to purified dCD46 protein.

To further analyse the role of *omp71* in RA-YM infectivity, an *omp71* gene deletion strain was constructed and verified by PCR (Additional file 2). To determine whether OMP71 is necessary for RA-YM binding to CD46, we incubated WT bacteria and the RA-YM Δ*omp71* strain with increasing concentrations of dCD46 protein. Both WT and RA-YM Δ*omp71* bound dCD46 in a dose-dependent manner, but the ability of the deletion strain to bind dCD46 at the same concentration was significantly lower than that of WT (Figure 4A). To further demonstrate that OMP71 interacted with dCD46, the direct binding assay analysed *E. coli*-expressing OMP71 at the surface. As the amount of dCD46 added was decreased, the amount of dCD46 bound to the *E. coli* OMP71 expression strain also decreased and was significantly higher than that of the corresponding empty vector strain (Figure 4B).

### Identification of OMP71 as the RA-YM adhesion protein

**Figure 5:**
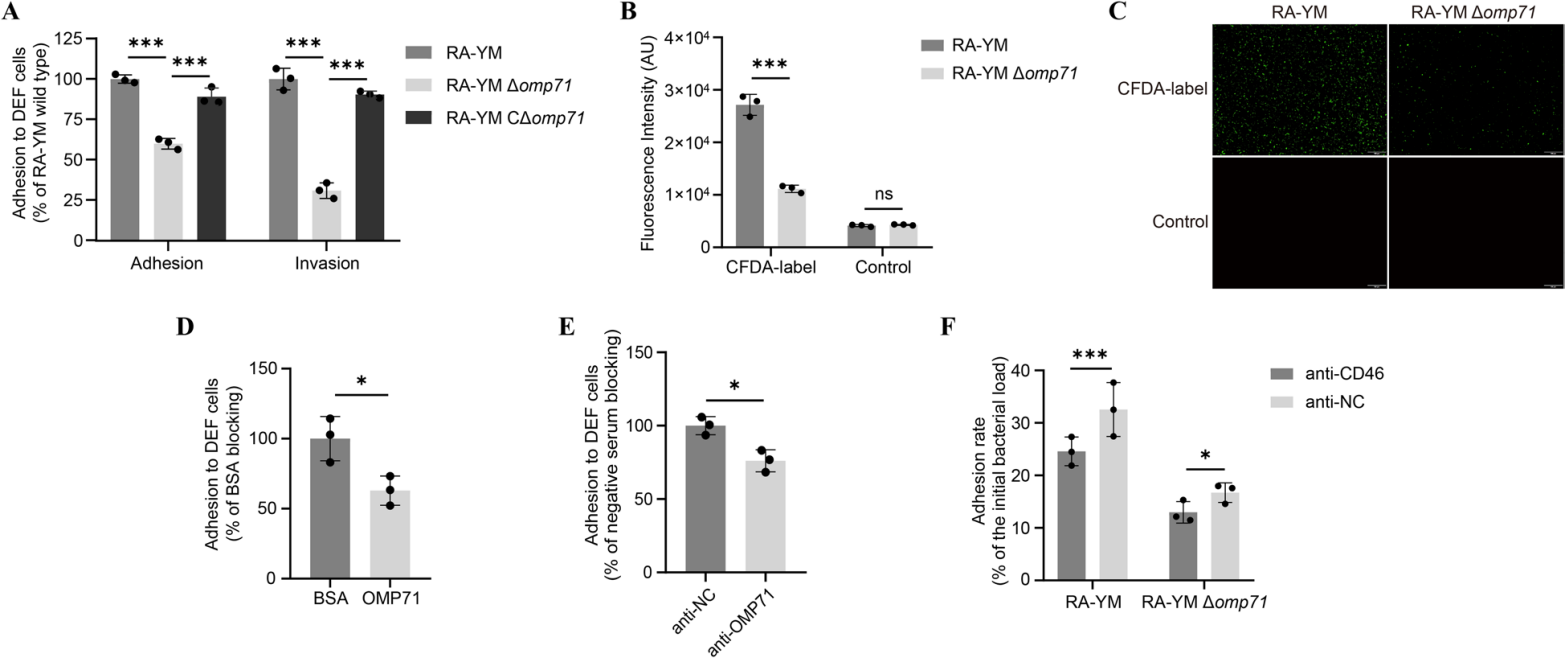
**Importance of OMP71 for RA-YM strain adhesion. A** Testing of the importance of OMP71 for RA-YM adhesion (*left*) and invasion (*right*) by counting of CFUs, as described [58]. RA-YM was set to 100%, and RA-YM Δ*omp71* internalisation of DEF is shown. For analysis of invasion, cells were subsequently treated with gentamicin to kill extracellular bacteria. The data were analysed using two-way ANOVA; error bars represent standard deviations. **B** and **C** CFDA-SE-fluor-labelled RA-YM and RA-YM Δ*omp71* adhered to DEFs for fluorescence intensity and fluorescence images, respectively, and unlabelled RA-YM and RA-YM Δ*omp71* were used as controls. The data were analysed using two-way ANOVA; error bars represent standard deviations. **D** and **E** Inhibition of RA-YM adhesion to DEF cells by the (**D**) purified OMP71 protein (*P < 0.05 compared with BSA group) and **E** anti-OMP71 polyclonal antibody (*P < 0.05 compared with the negative rabbit serum group). The data were analysed using one-way ANOVA; error bars represent standard deviations. **F** Inhibition of RA-YM and RA-YM Δ*omp71* adhesion by blocking CD46 on the surface of DEFs. All data are the mean of three experimental replicates, and data were processed by two-way ANOVA. Error bars represent standard deviations. *P ≤ 0.05, ***P ≤ 0.001.

To determine whether OMP71 was involved in RA-YM adherence to DEFs, RA-YM, RA-YM Δ*omp71*, and RA-YM CΔ*omp71* were incubated with DEFs for 2 h at 37 °C. After washing off the unbound bacteria, the bound cells were detached by trypsinisation, harvested by centrifugation, and plated on agar. The number of CFUs was counted. It was found that the amount of RA-YM Δ*omp71* strain bound to DEFs was 40% lower than that of the WT strain, while the amount of the complemented strain bound to DEFs was approximately 89% of that of the WT strain. These results show that OMP71 contributes to bacterial adherence to host cells; however, other membrane proteins also play a role in this process (Figure 5A, left).

To further establish if OMP71 was involved in the entering of DEFs by RA-YM, the bound bacteria were killed by incubation with gentamicin for 1 h. The number of viable RA-YM in the cells was determined, revealing that the invasion efficiency of RA-YM Δ*omp71* was approximately 70% lower than that of the WT strain, and the invasion efficiency of RA-YM CΔ*omp71* was only around 10% lower than that of the WT strain (Figure 5A, right).

When RA-YM and RA-YM Δ*omp71* were labelled with the CFDA-SE fluor and incubated with DEFs, fluorescence microscopy showed that more WT bacteria adhered to DEFs than the deletion strain (Figure 5B). The fluorescence intensity with the WT was approximately twice that of the deletion strain (Figure 5C). Moreover, pre-incubation of DEFs with OMP71 protein reduced the ability of RA-YM to adhere to DEFs (Figure 5D). Blocking OMP71 on the surface of RA-YM with anti-OMP71 polyclonal antibody equally decreased the ability of RA-YM to adhere to DEFs (Figure 5E). When DEFs were incubated with rabbit anti-dCD46 polyclonal antibody to block CD46 on the surface, the number of adherent RA-YM was significantly lower than that incubated with rabbit negative serum, with a slight difference observed in *omp71* deletion strains (Figure 5F). These results indicate that OMP71 is an important adhesin protein for RA-YM, while OMP71 mediates RA-YM adhesion to DEFs by binding to CD46.

### Knockout of *omp71* significantly reduced RA-YM pathogenicity

**Figure 6:**
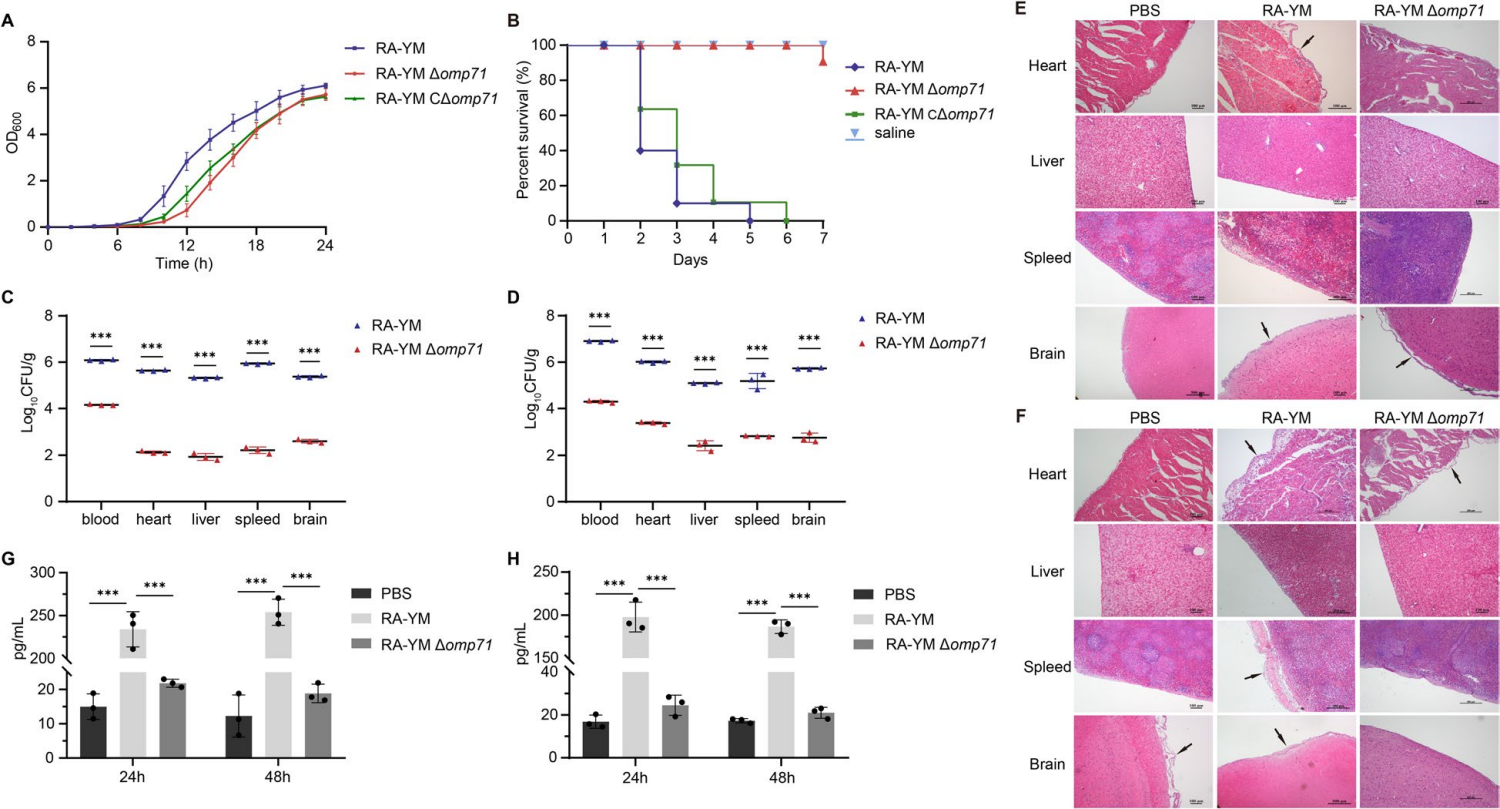
**Knockout of *omp71* resulted in reduced pathogenicity of RA-YM in ducklings. A** Determination of growth curve of RA-YM WT strain and *omp71* gene deletion strain. **B** Survival rate in ducklings infected by RA-YM and RA-YM Δ*omp71* strains. Ten twelve-day-old ducklings per group infected with 10^9^ CFU of bacteria were used to assess survival. **C** and **D** Bacterial loading of blood and tissues of ducklings 24 h and 48 h after bacterial infection. Twelve-day-old ducklings were infected with 10^6^ CFU of RA-YM and RA-YM Δ*omp71*. Blood, heart, liver, spleen, and brain were collected 24 h and 48 h later, and single colonies were counted after serial dilution and agar plating. **E** and **F** Histopathological analysis of ducklings infected with the RA-YM and RA-YMΔ*omp71* strains for 24 h and 48 h. **G** and **H** The levels of IL-6 (**G**) and IL-8 (**H**) in duck serum were determined by ELISA after 24 h and 48 h of bacterial infection. Statistical significance was assessed using a two-way analysis of variance (ANOVA). Error bars represent standard deviations from three independent experiments (***P < 0.001).

To explore the effect of *omp71* on the pathogenicity of RA-YM, we first obtained the growth curves of RA-YM, RA-YM Δ*omp71*, and RA-YM CΔ*omp71* (Figure 6A) and determined their pathogenicity in ducklings. The results indicate that all ducklings infected with the WT strain had died by the fifth day after the challenge, with the first deaths occurring on the second day (resulting in 100% mortality). The mortality rate of RA-YM CΔ*omp71* is comparable to that of the WT strain, but the time it takes for death to occur is slower. Ducklings infected with the same dose of RA-YM Δ*omp71* did not begin to die until the seventh day and had a mortality rate of only 10% (Figure 6B). The relative virulence of the two RA-YM strains was compared by measuring their LD_50_, and that for the Δ*omp71* mutant was 2.51 × 10^9^ CFU, which was 5.01 × 10^4^-fold higher than that of the WT RA-YM (5.01 × 10^4^ CFU).

The bacterial loads of WT and Δ*omp71* in the heart, liver, spleen, brain, and blood were measured to evaluate the effects of systemic infection of ducklings with RA-YM. The bacterial load of RA-YM Δ*omp71* was significantly lower than that of WT RA-YM in the heart, liver, spleen, brain, and blood 24 h and 48 h after challenge (Figures 6C and D). Histopathological examination of stained sections revealed that the level of tissue damage caused by the WT strain was significantly greater than with RA-YM Δ*omp71*.

Systemic WT infection after 24 h, manifested as mild epicardial oedema, mild liver steatosis, meningeal thickening with mild inflammatory cell infiltration, and symptoms of meningitis. In the RA-YM Δ*omp71* infection group, only a small amount of inflammatory cell infiltration was observed in the meninges, and other tissues showed no obvious pathological changes (Figure 6E). At 48 h after infection, there were fibrinous pericardial exudates in the WT RA-YM infection group, serous membrane thickening and oedema in the spleen, visible eosinophils accompanied by fibrinous exudates, a decrease in the number of lymphocytes in the spleen, and obvious inflammatory reactions in the meninges. There was mild epicardial oedema in the RA-YM Δ*omp71* group, but other organs showed no obvious pathological changes (Figure 6F).

Moreover, inflammatory factors related to blood in the in vivo environment are critical for evaluating the infection process. The levels of IL-6 and IL-8 in duck serum were noticeably higher in the RA-YM infection group after 24 and 48 h of challenge, compared to both the PBS group and the RA-YM Δ*omp71* infection group. The RA-YM Δ*omp71* infection group showed slightly higher levels of IL-6 and IL-8 than the PBS group. However, the difference was insignificant (Figures 6G and H).

## Discussion

Bacterial adhesion and colonisation on the surface of host cells is the fundamental initial step in infection. *R. anatipestifer* causes respiratory disease and is one of the most harmful pathogens infecting poultry [39], but its pathogenic mechanism is still not fully understood. Previous studies have shown that *R. anatipestifer* can adhere to and invade host cells [20]. Therefore, in this study, we aimed to determine the adhesion factors of *R. anatipestifer* and develop an understanding of the mechanism of bacterial host cell invasion with the goal of improving the prevention and control of *R. anatipestifer* infection.

Adhesion of pathogens to host cells is a complex process, usually involving a variety of adhesins and target receptors. Membrane-associated complement regulatory proteins protect the host from complement self-reaction and are often targeted by pathogenic microorganisms [40]. For example, the ubiquitous expression of CD46 on the cell surface and its regulatory activity on the complement system and cell signalling ability make it a likely target of various pathogens [41]. The OMPs of Gram-negative bacteria are directly involved in adhesion, colonisation, and persistent infection. In this study, we screened RA-YM’s OMPs to identify which ones interacted with dCD46. Through this process, we discovered OMP71, which enables RA-YM to adhere to host cells by binding to dCD46.

CD46 is an abundant complement regulatory factor on the cell surface and serves as a receptor for several pathogens [28, 42, 43]. It is closely associated with bacterial infection and immunosuppression caused by infection and has a crucial role in microbial immune escape and autoimmune diseases [44–46]. Like chicken CREM protein [27, 47], dCD46 is composed of five N-terminal complement control protein repeats (SCR1-5), an SCR-like domain, a transmembrane domain (TM), and a C-terminal cytoplasmic tail (CYT) located in the cell. In this study, a 1227 bp duck CD46 gene fragment was cloned by PCR, and the recombinant eukaryotic expression vector pFastBac-Strep-dCD46 was successfully constructed and transfected into Sf9 cells for eukaryotic expression.

Eleven candidate OMPs of *R. anatipestifer* were screened by Strep pull-down using purified dCD46 protein as bait and identified using mass spectrometric. A cold shock vector (pCold-TF) containing a 48 kDa molecular chaperone trigger factor [48] was fused with the target protein to increase the solubility of the OMPs. Notably, *R. anatipestifer* OMPs contain a large number of codons rarely found in *E. coli* that result in very low recombinant protein expression; however, Rosetta (DE3) can recognise six rare *E. coli* codons (AUA, AGG, AGA, CUA, CCC, and GGA) to improve the level of expression and the accuracy of the protein when translated in *E. coli* [49].

The following His_6_-tagged recombinant proteins were expressed and purified: RagA, OMP71, SusC, PorT, CLP, PNPLAs, OMP β-brl, HP, tbuX, DUF4353, and OprO. Despite the low concentration of purified protein obtained and the depletion of proteins by chaperone excision (results not shown), the trigger factor was still retained.

To further screen for interaction with dCD46, we verified the binding of OMP71 to dCD46 by ELISA and pull-down assays. The direct binding between the two proteins was verified by an in vitro pull-down assay. Western blotting confirmed that the binding ability of *R. anatipestifer* to dCD46 was decreased after *omp71* knockout compared with the WT strain. *E. coli* BL21(DE3)/pET-16b-*omp71* showed stronger binding to rdCD46 than *E. coli* BL21(DE3)/pET-16b in a dose-dependent manner. The results above determined that OMP71 is an important protein that binds CD46 to *R. anatipestifer*.

Moreover, the ELISA results indicated that CD46 may interact with other outer membrane proteins in addition to OMP71. Whether these proteins mediate the binding of CD46 to the bacteria needs to be substantiated. Similarly, more than one surface protein of *Haemophilus influenzae* (*H. influenzae*) can bind to complement regulatory factor. For example, its surface proteins E and F can bind to complement regulatory factor vitronectin and contribute to bacterial colonisation and infection [50, 51].

The OMPs found in Gram-negative bacteria, including *Helicobacter pylori* HopS, *Neisseria gonorrhoea* Opa, and *R. anatipestifer* OmpA, are commonly associated with bacterial adhesion and pathogenicity [18, 52–55]. An outcome of our study was that the number of bacteria adhering to DEF cells decreased by approximately 40% with the RA-YM Δ*omp71* mutant compared to WT. Moreover, the purified OMP71 protein was able to adhere directly to DEFs, and the OMP71 protein and anti-OMP71 polyclonal antibody were able to block the adhesion of RA-YM to DEFs (Figure 5). This result further indicates that OMP71 is an adhesion factor of *R. anatipestifer*. Additionally, the anti-dCD46 polyclonal antibody was able to block RA-YM adhesion to DEFs but not RA-YM Δ*omp71*, supporting the hypothesis that OMP71 binding to CD46 mediates *R. anatipestifer* adhesion to DEFs.

In the pathogenicity experiment of Cherry Valley ducks, the virulence of RA-YM Δ*omp71* decreased by 5 × 10^4^ times compared with the WT strain, suggesting that OMP71 is also an important virulence factor of *R. anatipestifer*, which is likewise a significant respiratory pathogen. Like *H. influenzae* [56], *R. anatipestifer* needs to cross the upper respiratory barrier, spread, and then survive in the blood before it can attack tissues and cause inflammatory reactions in tissues. The inflammatory response during infection in *H. influenzae* is determined by several virulence factors, including capsule, fimbriae, and OMPs [57]. In this study, infection with *R. anatipestifer* caused obvious inflammation in the heart, liver, spleen, and brain of ducklings. Deleting the *omp71* gene, which encodes virulence factors, led to the reduction or even disappearance of inflammatory reaction. Similarly, the contents of cytokines IL-6 and IL-8 in the blood of ducklings infected with *R. anatipestifer* increased greatly, but the increase was not significant after the deletion of *omp71*. It is suggested that the virulence factor of OMP71 may be fundamental to the inflammatory reaction caused by *R. anatipestifer* infection.

For agricultural animals, vaccination is the most effective measure to control infectious diseases. However, due to the complex serotypes of *R. anatipestifer* and the weak cross-protection between different serotypes, the application of inactivated vaccine has limited effectiveness. Additionally, the disease has a high incidence rate at the age of 2 to 3 weeks, during which the vaccine does not offer adequate protection. Therefore, antibiotic control has become the first choice in production, but it can cause increased antibiotic resistance and the emergence of new drug-resistant strains [58–61]. Currently, research on an *R. anatipestifer* vaccine has mainly focused on inactivated and attenuated vaccines. However, such a vaccine would not provide cross-protection for multiple serotypes of *R. anatipestifer*. Therefore, the exploration of OMPs of *R. anatipestifer* from the perspective of pathogen-host interaction lays a foundation for further study on the pathogenic mechanism of *R. anatipestifer* and the development of new subunit vaccines.

## Supplementary Information

**Additional file 1. Amplification of dCD46 gene and identification of its expression in Sf9 cells**. A The duck CD46 gene was amplified with a size of 1227bp. B PCR identification of recombinant positive baculovirus Bacmid-dCD46. Lanes: M, molecular weight marker; 1, 3: negative control; 2: Universal M13 tail identification Bacmid-dCD46; 4: Bacmid-dCD46. C The expression of Strep-dCD46 in Sf9 cells was detected by Western blot assay using anti-Strep-tag II antibody. Lanes: M, molecular weight marker; lane 1, supernatant of cell lysate; lane 2, precipitation of cell lysate; lane 3, culture medium supernatant. D The expression of Strep-dCD46 in Sf9 cells was detected by indirect immunofluorescence assay using a Mouse anti-Strep-tag II antibody and FITC-Goat anti-Mouse antibody. (a) Normal Sf9 cells; (b) Sf9 cells after transfection.

**Additional file 2. Construction of *R. anatipestifer*** **RA-YM Δ*omp71*** **and** **CΔ*omp71.*** A Amplification of the 5′ and 3′ homology arms of *omp71* and the spectinomycin resistance gene. Lanes: M, molecular weight marker; 1, 5′ homology arm; 3, *spec* gene; 5, 3′ homology arm; 2,4,6, negative control. B PCR identification of the *omp71* gene deletion strain. Lanes: M, molecular weight marker 1, *omp71* gene in RA-YM; 2, *omp71* gene in RA-YM Δ*omp71*; 3, *omp71* gene in *omp71*-LSR; 4, *spec* gene in *omp71*-LSR; 5, *spec* gene in RA-YM Δ*omp71*; 6, *spec* gene in RA-YM. C PCR identification of the *omp71* gene complement strain. Lanes: M, molecular weight marker 1, *omp71* gene in RA-YM; 2, *omp71* gene in RA-YM CΔ*omp71*; 3, *omp71* gene in RA-YM Δ*omp71*.

## Data Availability

The original contributions presented in the study are included in the article and Supplementary Materials. Further inquiries can be directed to the corresponding author. The mass spectrometry raw files have been deposited to ProteomeXchange with the accession number PXD052038.
