# Supplementary figures and images for: The outer membrane protein, OMP71, of Riemerella anatipestifer, mediates adhesion and virulence by binding to CD46 in ducks
Source: Vet Res. 2024 Oct 15;55:138. doi: 10.1186/s13567-024-01393-9 (PMC11481396; doi:10.1186/s13567-024-01393-9)

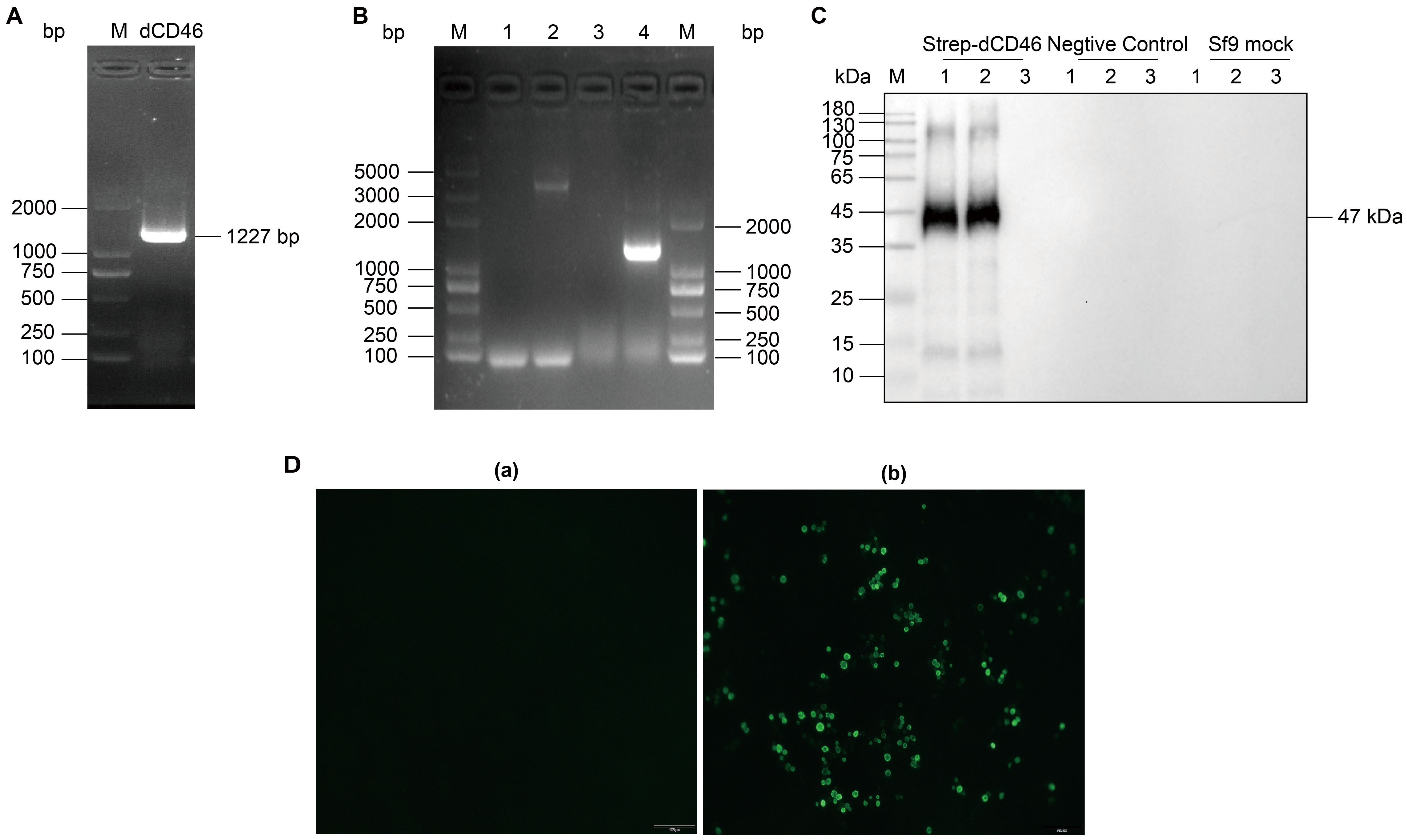

Supplement: Supplementary file 1 — Additional file 1. Amplification of dCD46 gene and identification of its expression in Sf9 cells. A The duck CD46 gene was amplified with a size of 1227bp. B PCR identification of recombinant positive baculovirus Bacmid-dCD46. Lanes: M, molecular weight marker; 1, 3: negative control; 2: Universal M13 tail identification Bacmid-dCD46; 4: Bacmid-dCD46. C The expression of Strep-dCD46 in Sf9 cells was detected by Western blot assay using anti-Strep-tag II antibody. Lanes: M, molecular weight marker; lane 1, supernatant of cell lysate; lane 2, precipitation of cell lysate; lane 3, culture medium supernatant. D The expression of Strep-dCD46 in Sf9 cells was detected by indirect immunofluorescence assay using a Mouse anti-Strep-tag II antibody and FITC-Goat anti-Mouse antibody. (a) Normal Sf9 cells; (b) Sf9 cells after transfection. [file 13567_2024_1393_MOESM1_ESM.png]

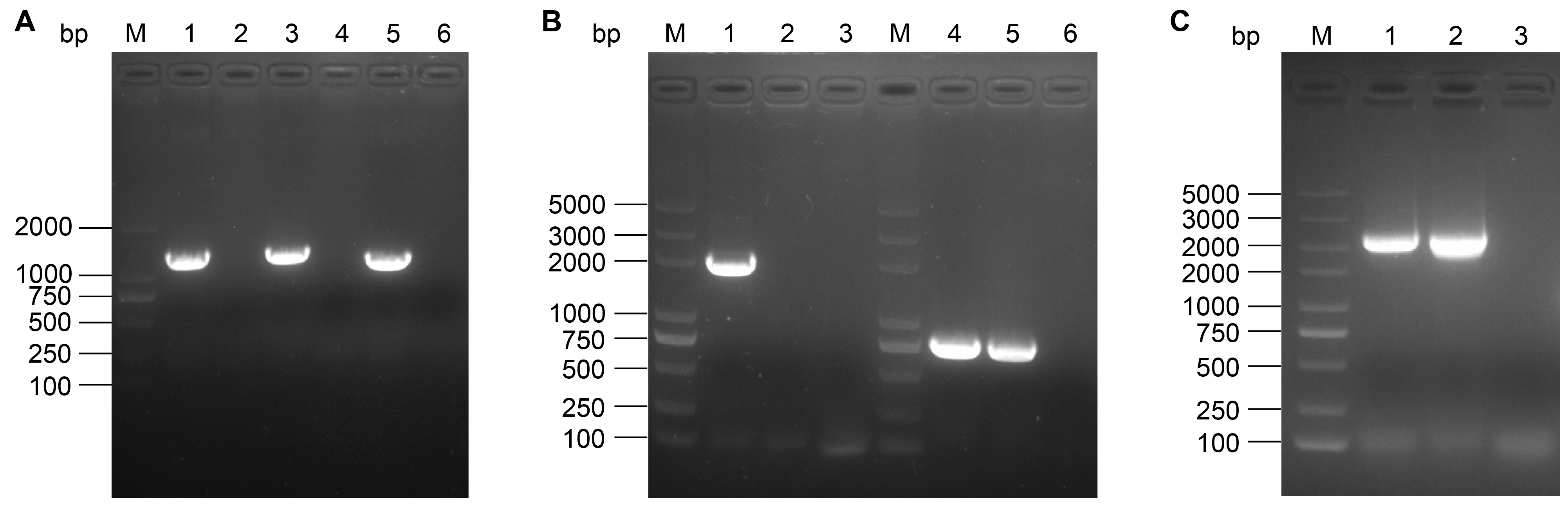

Supplement: Supplementary file 2 — Additional file 2. Construction of R. anatipestifer RA-YM Δomp71 and CΔomp71. A Amplification of the 5′ and 3′ homology arms of omp71 and the spectinomycin resistance gene. Lanes: M, molecular weight marker; 1, 5′ homology arm; 3, spec gene; 5, 3′ homology arm; 2,4,6, negative control. B PCR identification of the omp71 gene deletion strain. Lanes: M, molecular weight marker 1, omp71 gene in RA-YM; 2, omp71 gene in RA-YM Δomp71; 3, omp71 gene in omp71-LSR; 4, spec gene in omp71-LSR; 5, spec gene in RA-YM Δomp71; 6, spec gene in RA-YM. C PCR identification of the omp71 gene complement strain. Lanes: M, molecular weight marker 1, omp71 gene in RA-YM; 2, omp71 gene in RA-YM CΔomp71; 3, omp71 gene in RA-YM Δomp71. [file 13567_2024_1393_MOESM2_ESM.png]
